# Resolving mobile halide diffusion in perovskite films with frequency-domain photoluminescence

**DOI:** 10.1039/d6el00090h

**Published:** 2026-09-10

**Authors:** Sarah C. Gillespie, Agustin O. Alvarez, Bruno Ehrler, Veronique S. Gevaerts, L. J. Geerligs, Gianluca Coletti, Erik C. Garnett

**Affiliations:** a LMPV-Sustainable Energy Materials Department, AMOLF Institute Science Park 104 Amsterdam 1098XG The Netherlands e.garnett@amolf.nl; b TNO Department Solar Energy Westerduinweg 3 Petten 1755LE The Netherlands; c School of Photovoltaic and Renewable Energy Engineering, University of New South Wales Sydney New South Wales 2052 Australia; d University of Amsterdam Science Park 904 Amsterdam 1098XH The Netherlands

## Abstract

Intensity-modulated photoluminescence spectroscopy (IMPLS) is a frequency-domain, fully optical technique that probes ionic processes in halide perovskites. Here, IMPLS is expanded to resolve photoluminescence (PL) spectra, instead of only the emission intensity, as a function of excitation modulation frequency. For a triple-cation, mixed-halide perovskite, the PL peak energy and bandwidth are tracked between 2 mHz and 1 Hz, revealing distinct spectral responses. To identify the halide species governing these responses, IMPLS is further measured on four simplified compositions, MAPb(I_*x*_Br_1−*x*_)_3_ where *x* = 1, 0.8, 0.5, 0. The results show that the pure-bromide sample exhibits a distinctly different response, with an onset at a lower frequency. The onset frequency differences between the pure-bromide and pure-iodide samples align with diffusion coefficient differences reported in literature. These findings demonstrate the benefits of spectrally resolving IMPLS – providing chemical insight into reactive species and better interpretation through combined spectral and intensity analysis.

## Abstract 2

Intensity-modulated photoluminescence spectroscopy (IMPLS) is a frequency-domain, fully optical technique that probes ionic processes in halide perovskites.

## Graphical abstract

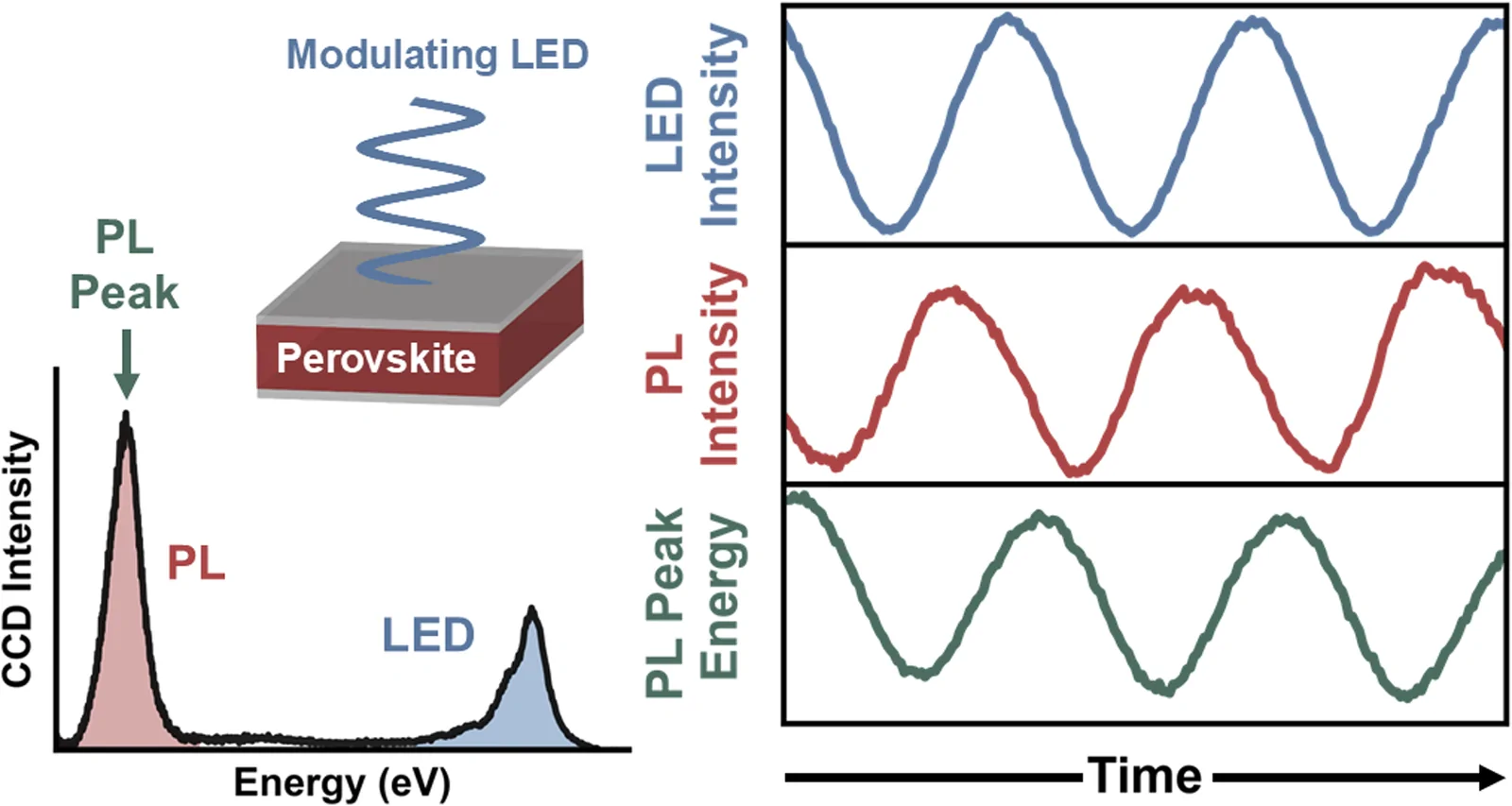

**Broader context**

The widespread commercialization of halide perovskites in photovoltaic applications remains limited by the intrinsic instability of the perovskite layer itself. This instability primarily arises from ion migration and halide redistribution within the films under illumination and electrical bias. Understanding these processes is therefore essential for improving both device stability and efficiency. Here, we demonstrate that intensity-modulated photoluminescence spectroscopy (IMPLS) can spectrally resolve frequency-dependent photoluminescence responses associated with mobile halide processes in perovskite films. The spectral responses are distinct from the corresponding intensity response, indicating that additional chemical and compositional information can be resolved in the frequency domain. We further show that the dominant low-frequency response is primarily associated with halide-related transport, with onset frequencies consistent with reported differences in halide diffusion coefficients. These findings establish spectrally resolved IMPLS as a contact-free approach for optically quantifying mobile ionic phenomena in perovskites.

## Introduction

Ion migration is a defining characteristic of halide perovskites that strongly influences their optoelectronic properties.^1^ Due to their low migration energies, mobile ionic defects can readily redistribute across the perovskite layer under even moderate electrical bias or illumination conditions.^2,3^ These defects can accumulate at interfaces and chemically react with charge transport layers or screen the electric field, which can alter both carrier extraction efficiencies and recombination rates over time.^4,5^ For solar cells in particular, while extrinsic degradation pathways, such as those induced by moisture or oxygen, can largely be mitigated through encapsulation, the intrinsic instability associated with ion migration remains a challenge.^1,6–8^ At the same time, ionic redistribution is not always detrimental. When understood and controlled, it can enable novel optoelectronic functionalities, including optical memory states in devices and self-tracking mechanisms in solar concentrators.^9–11^ Whether the goal is to mitigate ionic effects to enhance stability or to exploit them for new technologies, it is essential to fundamentally understand mobile ionic processes in perovskites.

Electrical methods – such as impedance spectroscopy (IS) – have been employed to quantify mobile ionic properties such as activation energy and diffusion coefficient in complete devices.^12–19^ In IS, a small sinusoidal electrical perturbation is applied to the device, and the resulting response is measured as a function of frequency.^18,20,21^ Typically, the perturbation is introduced through a voltage modulation while the current response is recorded, although the inverse configuration is also possible.^20^ The response is characterized by both the amplitude of the current |*Ĩ*| and the relative phase shift *θ* between the applied and measured oscillating signals. Together with the voltage amplitude |*Ṽ*|, the impedance transfer function is defined as1
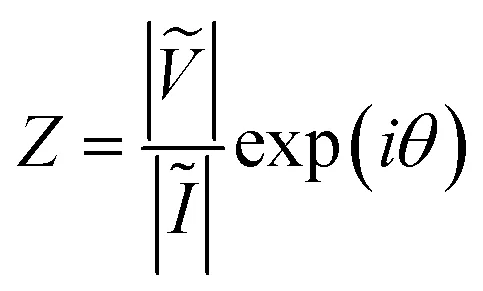


from which the real and imaginary components of the impedance can be extracted and represented in Bode or Nyquist plots. Distinct processes with characteristic relaxation times can be resolved across the frequency range; these processes are then physically interpreted using representative equivalent circuit (EC) models or drift–diffusion simulations.^16,22,23^ IS can be performed under different offset voltages, temperatures, and illumination conditions, and is often combined with corresponding transient methods. Using such approaches, electronic and ionic diffusion lengths, ion activation energies and defect densities have been derived.^12,14,16,24,25^

Despite the benefits of IS for perovskite device characterization, the interpretation of ion-related IS features remains challenging. This difficulty arises from several factors, including different Nyquist spectral features that can be obtained on similar stacks, device degradation during the measurement (often resulting in loops in the Nyquist spectra), interfacial ionic reactions and recombination at the transport layers.^23,26,27^ The interfacial effects in particular can dominate the IS signal and obscure fundamental ionic processes.^28,29^ In response to these challenges, several studies have provided essential guidelines for ensuring that IS measurements are both correctly performed and meaningfully interpreted.^18,20,22,23,30,31^ Nevertheless, the need for electrical contacts imposes inherent limitations on IS and indeed on all other electrical methods.

**Fig. 1:**
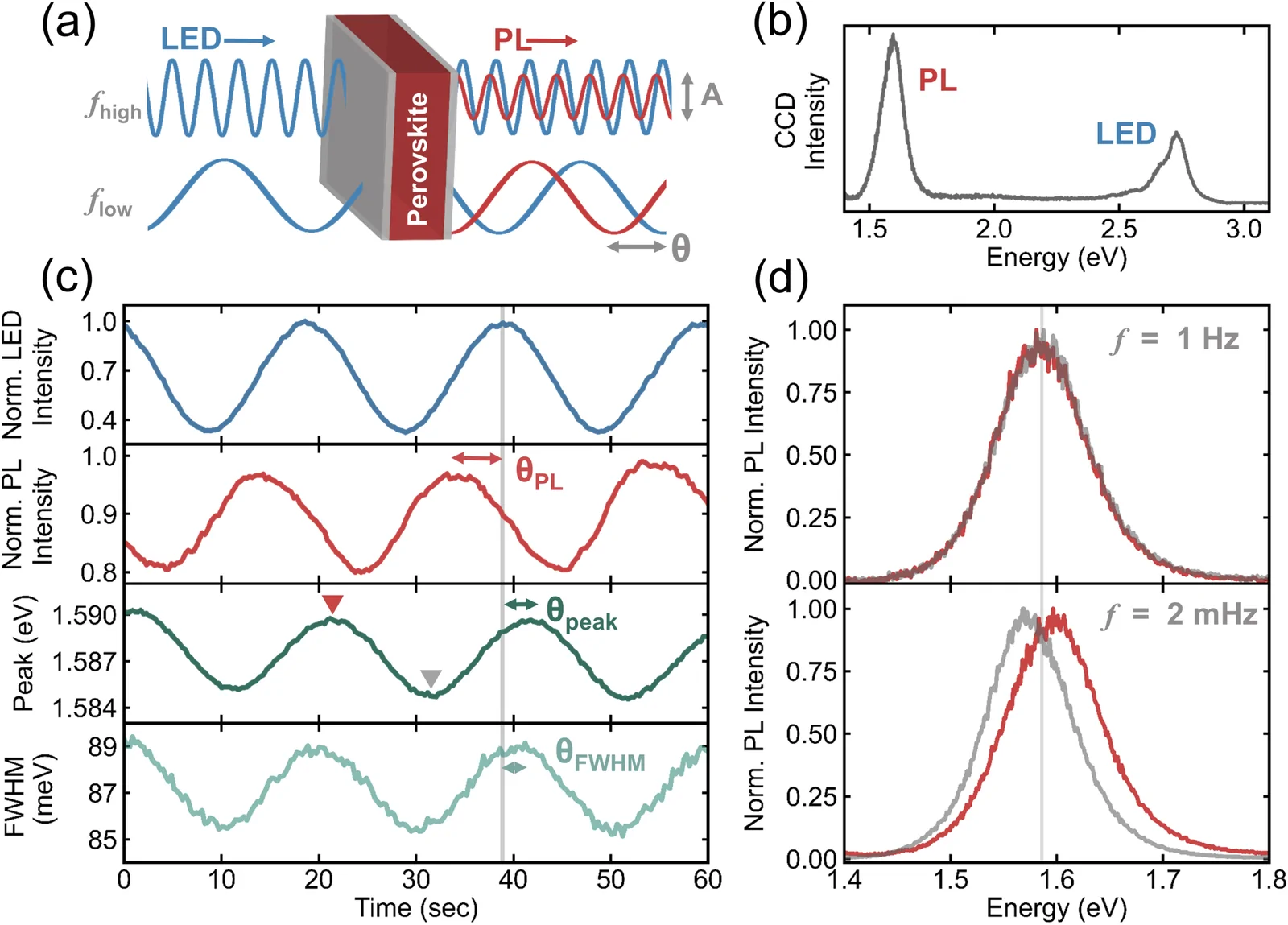
(a) Schematic of IMPLS: optical excitation is sinusoidally modulated by a 450 nm LED source (blue), and both the PL (red) and LED transmission signal from the perovskite sample are monitored. The PL response, measured as the phase shift (*θ*) and amplitude (*A*), varies with modulation frequency, as illustrated here by the relatively stronger phase shift and larger amplitude at *f*_low_ compared to *f*_high_. (b) Representative spectrum collected at a single time point (235 ms integration time), showing resolvable, non-overlapping PL and LED peaks across the spectral range. (c) Time series at *f* = 50 mHz over three periods, showing the integrated LED intensity (blue), PL intensity (red), PL peak energy (dark green) and FWHM (light green). The solid gray line marks a timestamp where the LED intensity is at its maximum. This line is extended across all four sub-panels as a visual guide. (d) Comparison of the PL spectral trends at *f* = 1 Hz (top) and *f* = 2 mHz (bottom). Red spectra correspond to the most blue-shifted state, while gray spectra represent the most red-shifted state. These locations are indicated by the corresponding color-coded triangular markers over the peak energy signal in Fig. 1c.

In response to this limitation, we recently applied intensity-modulated photoluminescence spectroscopy (IMPLS) as an optical analog to IS.^32^ In IMPLS, the modulated photoluminescence (PL) response is monitored under sinusoidal optical excitation, where the IMPLS transfer function is defined as2
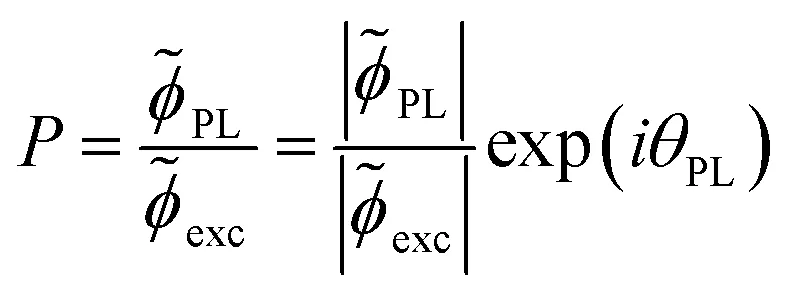
Here, *θ*_PL_ is the phase shift between the excitation and PL, and |*ϕ̃*_PL_|/|*ϕ̃*_exc_| is the ratio of PL to excitation intensity amplitudes (Fig. 1a). This transfer function has a similar mathematical form to the IS transfer function in eqn (1), as both describe a complex frequency-dependent response defined by the ratio between a measured response and an external perturbation. This allows frequency-domain analysis procedures and Nyquist representations to be applied to IMPLS data.^32^ However, the similar mathematical form does not imply that features in the optical transfer functions have the same physical meaning as capacitive or inductive elements in IS. Both IMPLS and IS offer complementary perspectives on material properties. For instance, IMPLS can be performed on perovskite films without electrical contacts, thereby avoiding complications introduced by charge transport layers. IMPLS enables an expanded set of measurement strategies, including full-field and localized point illumination conditions, and the response can be spatially mapped across the sample surface.^32,33^

However, unlike IS, IMPLS on contact-free films is restricted to open-circuit voltage (*V*_OC_) measurement conditions. In addition, using IMPLS to understand properties of mobile ions can be challenging, since the PL response is not exclusive to these ionic processes. Specifically, both the PL intensity and spectral shape of any thin-film semiconductor are inherently sensitive to numerous processes and material properties, including the band gap, temperature, film thickness, electronic carrier diffusion and carrier recombination.^34–36^ The measured spectral shape is also affected by optical effects such as reabsorption, interference, and outcoupling.^36^ Halide perovskite semiconductors introduce additional complexity, as their PL intensity and spectral shape can also be influenced by mobile-ion redistribution, chemical reactions, and interactions with the surrounding atmosphere.^37–39^ Moreover, many of these processes are inherently coupled. For example, light-induced halide segregation may involve halide oxidation, chemical reaction kinetics, and the diffusion of mobile halide species through the film and across grain boundaries.^40,41^ Similarly, the density and occupation of electronic traps can influence both carrier recombination and halide-segregation dynamics, while these dynamics may depend strongly on whether the sample is encapsulated or exposed to the surrounding atmosphere.^38^ The interpretation of an IMPLS response therefore requires some prior knowledge of the characteristic timescales of the relevant processes under the applied experimental conditions. Plausible alternative processes should also be considered and, where possible, ruled out.

Despite these interpretive challenges, the spectral sensitivity of IMPLS represents an important advantage over IS. For halide perovskites in particular, PL spectral features – specifically tracking the PL peak energy – has served as a proxy for visualizing photoinduced halide phase segregation; both the extent and kinetics of segregation can be monitored through PL time-series measurements.^38,39,41–43^ While some works recognize the technological novelty of halide segregation, its presence is generally regarded as detrimental to perovskite PV stability.^9,44,45^ PL time-series analysis – and IMPLS as the frequency-domain counterpart – provides compositional information linked to material performance that is inaccessible to purely electrical characterization approaches.

In this work, we demonstrate that frequency-domain responses of the PL spectra can be resolved using IMPLS. We quantify the frequency dependence of the PL peak energy and bandwidth (full width at half maximum, FWHM) for the same triple-cation, mixed-halide (TCMH) perovskite studied in our previous work.^33^ We further show that the spectral components can be visualized using Bode and Nyquist representations. To confirm that the frequency-dependent spectral features originate from halide-specific processes, we then compare the IMPLS responses of four simplified perovskite compositions with varying halide ratios. Importantly, we resolve distinct features which we attribute to different halide contributions, with the pure-bromide sample showing minimal response across the measured frequency window. This work highlights the simplicity of IMPLS as a powerful characterization method for probing and differentiating ionic processes in halide perovskites.

## Results and discussion

### Frequency-domain response of the PL spectrum

IMPLS was measured on a SiO_2_-encapsulated perovskite film with the composition Cs_0.07_(FA_0.8_MA_0.2_)_0.93_Pb(I_0.8_Br_0.2_)_3_. Offset (DC) excitation was provided by a 405 nm laser and the excitation modulation was provided by a 450 nm LED. All fabrication and measurement details are listed in the SI, while a schematic of the setup used is found in our earlier work.^33^ An example of a single complete spectrum is presented in Fig. 1b. The LED intensity is lower than the PL intensity because the measurement was conducted in transmission mode and most of the incident LED light was absorbed by the perovskite film. The absorptance was measured to be *A* = 70%.^33^

All spectra were fit to Lorentzian profiles, which enabled us to quantify the intensity variations due to the excitation modulation over time. To minimize the influence of initial phase segregation, all frequency-domain analyses were conducted only after the sample had been illuminated for at least 100 s, by which time the PL response had stabilized (Fig. S2).^46^ Representative LED (blue) and PL (red) intensity signals after this stabilization time are shown for three periods at *f* = 50 mHz in the upper two sub-panels in Fig. 1c. The fitting procedure additionally extracted the PL peak energy and the FWHM (third and fourth sub-panels in Fig. 1c). As shown in Fig. S3, the linearity of these responses under sinusoidal excitation was assessed using Lissajous plots, which exhibited undistorted elliptical trajectories.^20^ By comparing the four traces, the relative phase shifts of the peak energy (*θ*_peak_) and of the FWHM (*θ*_FWHM_) are distinct from the PL intensity phase shift (*θ*_PL_). This distinction provides preliminary evidence that the spectral components of the IMPLS response may contain information related to chemical or compositional changes, in addition to that obtained from the integrated PL intensity.

The frequency dependence of the PL spectral shape is illustrated by comparing the spectra at the measurement limits of *f* = 1 Hz and *f* = 2 mHz (Fig. 1d). The gray traces correspond to the most red-shifted peaks and the red traces to the most blue-shifted ones. At 1 Hz, the PL spectra show minimal variation across the modulation cycle, whereas at 2 mHz, the spectral shape exhibits pronounced oscillations. Further examples of the frequency-dependent spectral responses are presented in Fig. S4 in the SI.

**Fig. 2:**
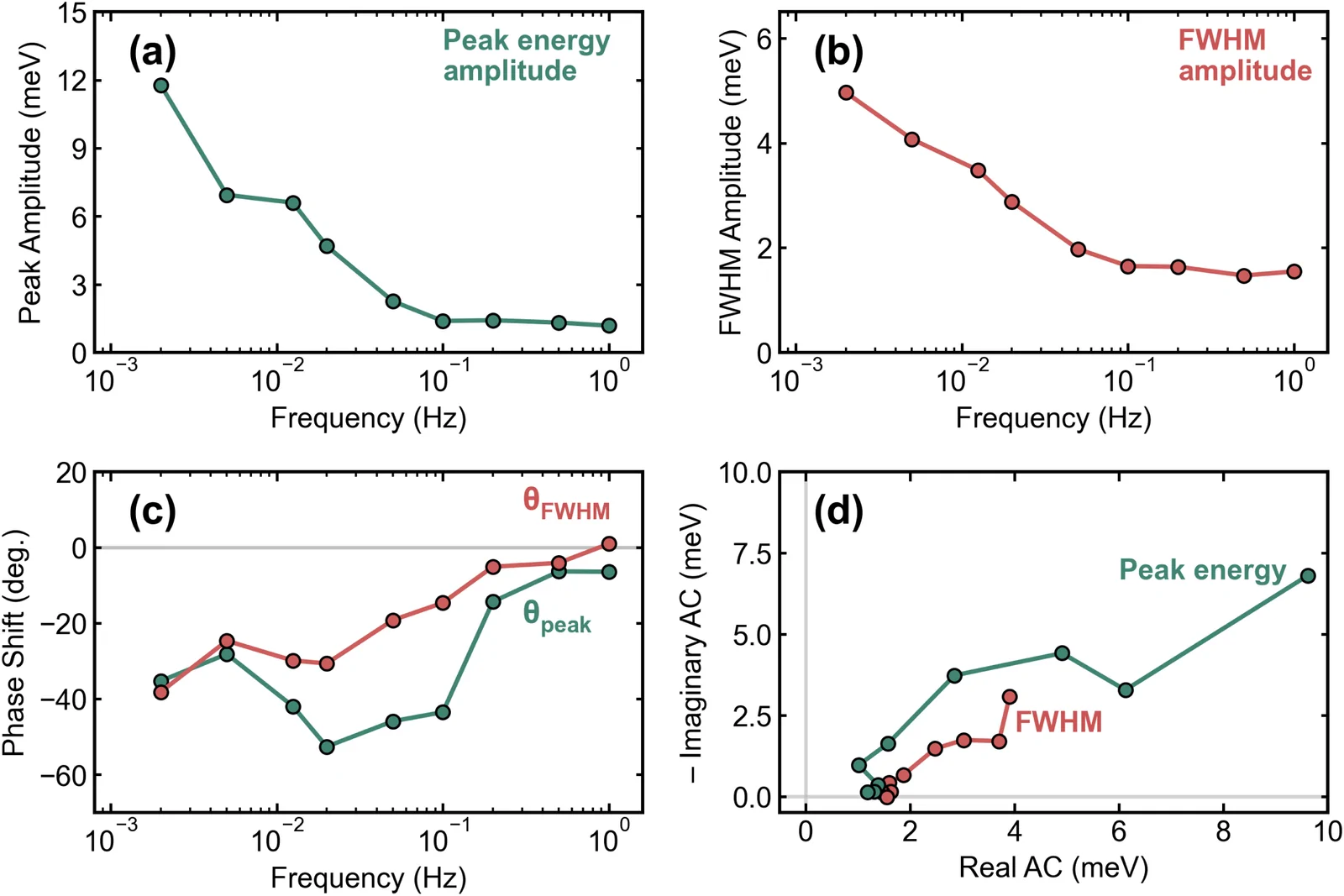
Amplitude Bode plots of (a) the PL peak energy and (b) the FWHM, measured between modulation frequencies of *f* = 1 Hz to *f* = 2 mHz. (c) Phase Bode plots for the same peak energy (green) and FWHM (red). (d) Corresponding Nyquist plots of the data, calculated using eqn (3) and (4) for the peak energy and FWHM, respectively.

In Fig. 2a and b, the absolute changes in the peak energy amplitude and FWHM amplitude are presented in Bode plots. The corresponding DC offset values are presented in Fig. S5 of the SI. Between *f* = 1 Hz and 100 mHz, both amplitudes remained constant; at lower frequencies, both increased. This inflection point corresponds to a process onset time of *τ*_onset_ = 1/2π*f* = 1.6 s. Fig. 2c further shows the phase shifts of the peak energy and FWHM relative to the LED intensity. These lagged behind the LED modulation, whereas the PL intensity – as shown in our earlier work – led the LED.^33^

Based on the increasing phase and amplitude trends for reducing modulation frequency, it is unlikely that these variations were solely from thermal influences. In a purely thermal model with the substrate as the limiting layer, the thermal diffusivity of glass (*α* ≈ 8 × 10^−3^ cm^2^ s^−1^) and thickness of the substrate (*L* = 1 mm) yield a characteristic thermal diffusion time constant of *τ*_therm_ = *L*^2^/*α* ≈ 1 s.^47^ Thus, for modulation frequencies below 1 Hz, the spectral responses would be expected to saturate. However, we observed the opposite, indicating that thermal diffusion alone cannot account for these trends.

To visualize the spectral responses using Nyquist plots, we defined the transfer function of the peak energy, Pe, relative to the LED excitation as3
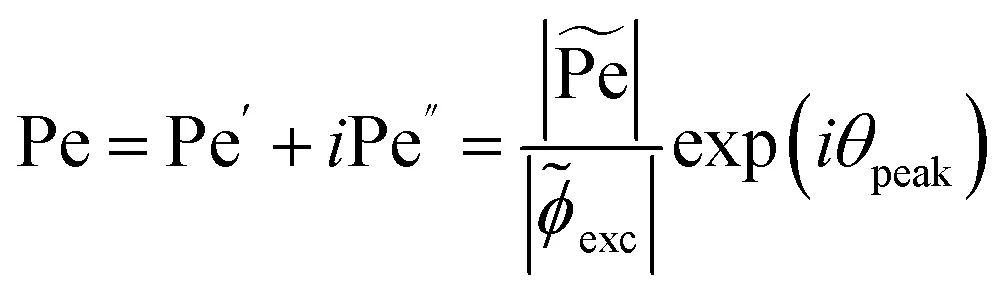
and the FWHM transfer function, *F*, as4
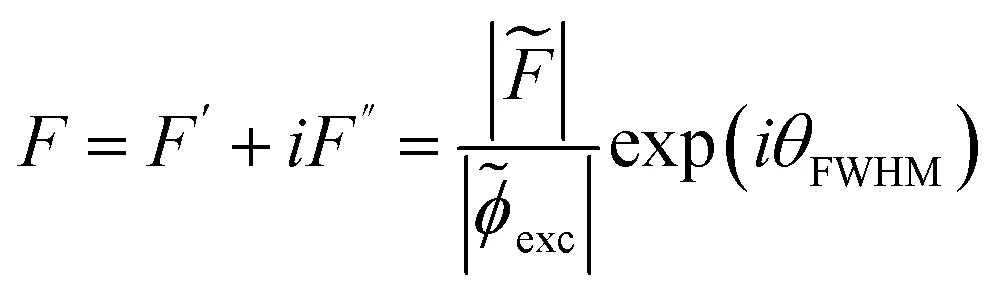


Since the LED amplitude remained constant across the frequency window during the measurements, we set |*ϕ̃*_exc_| = 1. This excitation normalization preserves the absolute energy scale in the Nyquist plots (Fig. 2d), but corresponds to an arbitrary excitation flux. The absolute magnitudes of the *F* and Pe responses therefore depend on the chosen excitation level and the DC offset operating point about which the system is perturbed. By inspecting the peak energy and FWHM arcs, the spectral responses exhibited a negative phase shift, resulting in features located in the first quadrant of the Nyquist representation. In contrast, the PL intensity IMPLS response exhibited a positive phase shift, resulting in a feature located in the fourth quadrant. In IS, capacitive and inductive elements can generate responses in the first and fourth quadrants, respectively. In our previous work, we have shown that the PL intensity IMPLS response can be described using electrical elements within an optical equivalent circuit framework.^32^ However, further work is required to fully understand the physical meaning of these features in the different IMPLS transfer functions and their possible relationship with the features observed in IS.

### IMPLS analysis on MAPb(I_*x*_Br_1−*x*_)_3_ films

Two key questions that follow from the spectral analysis are: what specifically governs the spectral response, and why does the IMPLS spectral element appear, to some extent, decoupled from the intensity element? Since the PL spectrum is partially influenced by the local halide composition, it is possible that halide segregation effects play a role, with defect-driven processes further modifying both the spectral and intensity features.^15,38,42,48^ However, the measured emission does not necessarily provide a direct representation of the overall halide composition or phase distribution, since photogenerated carriers can funnel into lower-band-gap iodide-rich regions.^49^ At the same time, the PL peak is not necessarily fixed at a single segregated-phase energy. Different excitation conditions can produce a broad range of emission wavelengths, consistent with the formation of different segregated states.^11^ Given the additional complexity of the triple-cation, mixed-halide composition, it is therefore challenging to attribute the observed responses solely to the halide distribution. Thus, to investigate whether the IMPLS response depends systematically on the X-site composition, we measured pure-iodide, pure-bromide, and two mixed-halide perovskites. To reduce the A-site complexity, we focused solely on methylammonium-based films, MAPb(I_*x*_Br_1−*x*_)_3_, with *x* = 1, 0.8, 0.5, 0. This ensured that the PL response was not obscured by mixed-cation effects.^50^

### Localized spot high-intensity results

**Fig. 3:**
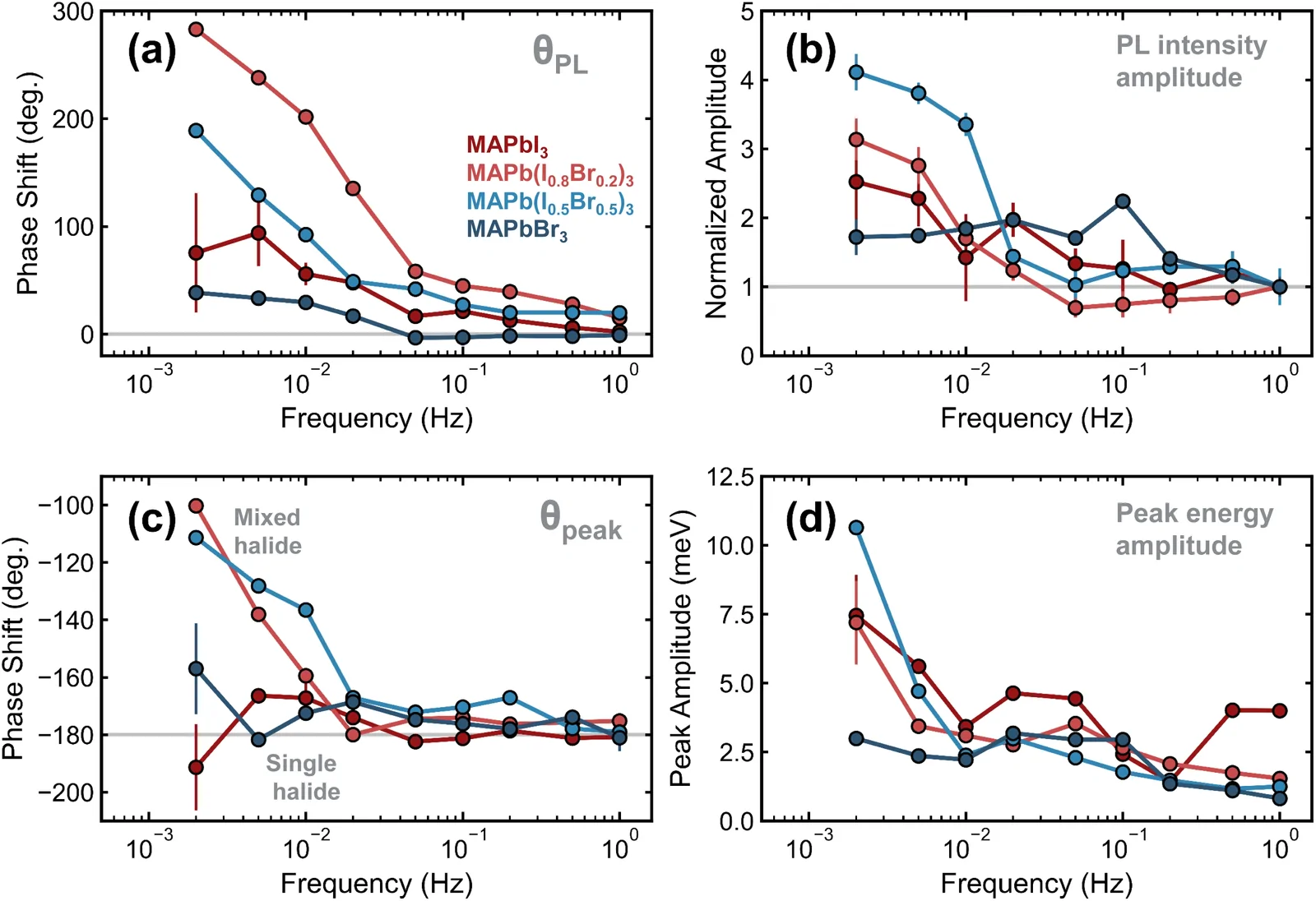
IMPLS results for the four MAPb(I_*x*_Br_1−*x*_)_3_ compositions, measured using both the 405 nm laser and 450 nm LED as excitation sources. MAPbI_3_ is shown in dark red, MAPb(I_0.8_Br_0.2_)_3_ in light red, MAPb(I_0.5_Br_0.5_)_3_ in light blue, and MAPbBr_3_ in dark blue. (a) Integrated PL intensity phase shift (*θ*_PL_) and (b) integrated PL intensity amplitude. The increasing amplitude and large phase shifts for reducing frequency are generally consistent with the TCMH perovskite response presented in our previous work.^33^ (c) Peak energy phase shift and (d) peak energy amplitude for the same samples. Error bars are shown for all data points and represent the combined experimental variation and fitting uncertainty.

We repeated the IMPLS experiments on the MAPb(I_*x*_Br_1−*x*_)_3_ samples to compare with the TCMH results shown in Fig. 2. The laser intensity was reduced to prevent sample degradation during these experiments (see SI for further experimental details). Fig. 3a and b show the IMPLS response of the integrated PL intensity for the four compositions: as the modulation frequency decreased, both the phase and amplitude increased. Broadly, the mixed-halide samples exhibited stronger IMPLS responses compared to the pure-halide samples. This is consistent with the corresponding PL time-series behavior as shown in Fig. S6 and S7. The enhanced response of the mixed-halide samples is consistent with contributions from halide segregation and the higher defect densities previously reported in mixed-halide films, although other coupled optical and electronic processes may also influence the measured PL response.^15,42,48^

The corresponding IMPLS trends of the peak energy phase shift and amplitude are shown in Fig. 3c and d. Additional spectral components are presented in Fig. S8. Unlike the TCMH peak energy response (Fig. 2c), all MAPb(I_*x*_Br_1−*x*_)_3_ samples exhibited peak energies nearly perfectly out of phase at frequencies above approximately 20 mHz. This behavior is attributed to a small photothermal effect: periodic heating induces bandgap shifts and, depending on whether the bandgap increases or decreases with temperature, the response can appear either perfectly in phase or perfectly out of phase with the excitation. At lower frequencies, the mixed-halide compositions diverged in their phase behavior compared to the pure-halide samples. This divergence is consistent with the spectral behavior of the mixed-halide systems, including TCMH, being influenced by interactions between the two halide species. However, because the PL spectrum may also be affected by carrier funneling, defects, reabsorption, and other coupled processes, this observation alone does not uniquely identify the underlying mechanism. Taken together with the composition dependence and characteristic frequency range, these results are consistent with halide segregation contributing strongly to the spectral response, whereas mobile-ion transport appears to contribute more strongly to the intensity response.^42^

### Full-field illumination results

**Fig. 4:**
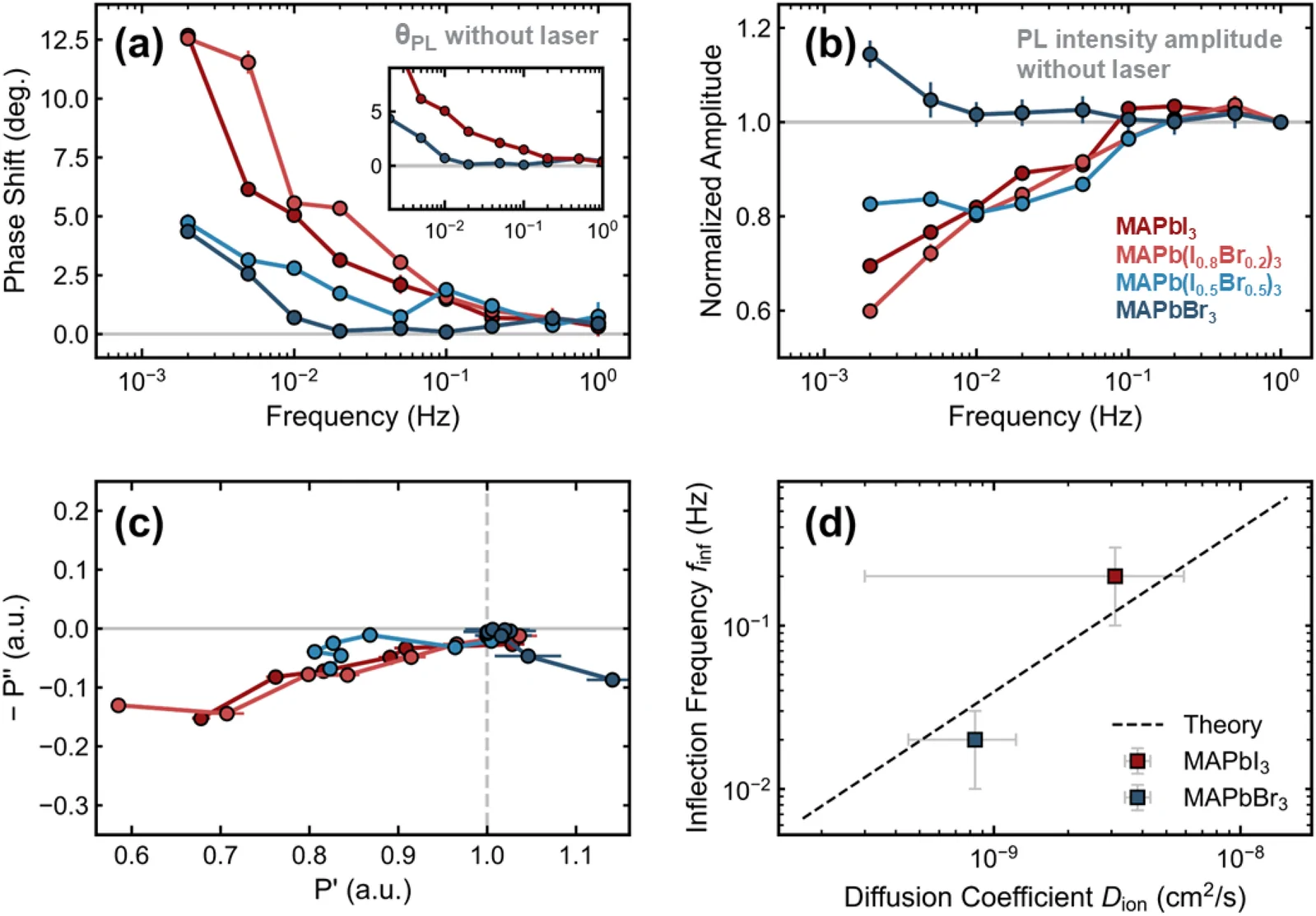
IMPLS results for the four MAPb(I_*x*_Br_1−*x*_)_3_ compositions, measured using only the 450 nm LED as the excitation source (without additional laser excitation). (a) Integrated PL intensity phase shift and (b) PL intensity amplitude. The pure-bromide sample exhibits a response onset frequency approximately one order of magnitude lower than that of the pure-iodide sample, as shown in the inset of panel a. The pure-bromide sample additionally does not exhibit amplitude quenching at reduced frequencies, unlike the mixed-halide and pure-iodide perovskite samples. (c) Corresponding IMPLS Nyquist plots, generated from the data in panels a and b, and using eqn (2). (d) Comparison between literature values of the halide diffusion coefficients for MAPbI_3_ and MAPbBr_3_ with the corresponding inflection frequencies determined in this work.^51,52^ The dashed black line represents the fit of the data, under the constraint that the slope *m* = 1, consistent with the theoretical expectation that *D*_ion_ ∝ *f*_char,diff_ ∝ *f*_inf_.

To further investigate these responses, we simplified the experimental conditions and removed the high intensity laser. We measured IMPLS on the MAPb(I_*x*_Br_1−*x*_)_3_ samples under full field, low intensity illumination using only the LED. This enabled direct comparison with the intensity response of the TCMH perovskite in our full-field analysis work.^32^Fig. 4a and b show the PL intensity phase shifts and amplitudes from the full field illumination experiment. Comparing these intensity results with those for the TCMH composition shown in our previous work, we found that the pure-iodide and mixed-halide perovskites exhibited similar phase and amplitude trends.^32^ Specifically, as the frequency decreased, the PL amplitude was reduced for all three iodide-containing perovskite films, consistent with the behavior of the TCMH perovskite. In contrast, the PL amplitude of MAPbBr_3_ remained nearly constant across the frequency range, aside from the slight enhancement at *f* ≲ 20 mHz. The onset of this process in MAPbBr_3_ occurred at a frequency approximately one order of magnitude lower than that of MAPbI_3_ (10–20 mHz *versus* 100–200 mHz, depending on whether the onset frequencies are read from the phase or amplitude plots). For clarity, the phase components of only MAPbBr_3_ and MAPbI_3_ are shown in the inset in Fig. 4a. We previously ascribed the process observed in the millihertz range to the diffusion of mobile halide species across the film thickness.^32^ Here, however, because the Nyquist arcs do not close within the measured frequency range (Fig. 4c), it is likely that mobile ions have not traversed the full film thickness even at the lowest frequency point. Nevertheless, their stretched shape is consistent with a distributed diffusive response, even if the ions redistribute over shorter distances during each modulation cycle.^20^ Stretched Nyquist arcs are commonly associated with transport processes, corresponding to the Warburg-like behavior in IS.^14,20^ This differs from the more ideal semicircular response expected for a single first-order relaxation process, such as defect-pair recombination, and therefore supports ionic diffusion as the dominant origin of the low-frequency feature. As elucidated in our earlier work, this ionic redistribution may be driven by internal electric-field gradients arising from differences between the surface and bulk trap-state densities or from the order-of-magnitude difference of mobilities between electrons and holes.^32,53^ These comparisons under full-field illumination therefore support an interpretation in which the measured IMPLS response is associated with halide-related transport and strongly depends on the halide composition of the sample, with iodide-containing samples exhibiting stronger responses compared to the pure-bromide samples.

Since the ionic diffusion coefficient is proportional to the characteristic diffusion frequency, *D*_ion_ ∝ 1/*τ*_char,diff_ ∝ *f*_char,diff_, and assuming the same mechanism for the MAPbI_3_ and MAPbBr_3_ samples, the difference in onset frequencies is consistent with diffusion coefficients previously reported by our research groups using transient ion-drift measurements: *D*_ion_ = (3.1 ± 2.8) × 10^−9^ cm^2^ s^−1^ for MAPbI_3_ and *D*_ion_ = (8.4 ± 3.9) × 10^−10^ cm^2^ s^−1^ for MAPbBr_3_.^51,52^ However, broader literature reports *D*_ion_ values spanning many orders of magnitude, depending on sample microstructure and stoichiometry, measurement conditions, and extraction techniques.^3,14,16,54–57^ Since these two values were obtained using the same measurement approach on samples prepared using closely comparable procedures and in the same chemical laboratory, we use them here as the most directly comparable values, rather than as representative of the full reported range. On this basis, Fig. 4d compares the measured inflection frequencies (assuming *f*_inf_ ∝ *f*_char,diff_) with the corresponding diffusion coefficients. The fixed-slope (*m* = 1) fit yields an intercept *b* corresponding to a proportionality constant 10^*b*^ = 3.9 × 10^7^. Since5
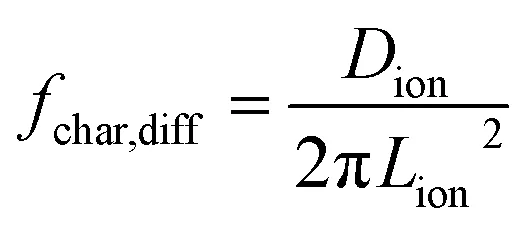
where *L*_ion_ is the ionic diffusion length, and setting *β* as the proportionality constant between the inflection and characteristic frequencies, then6
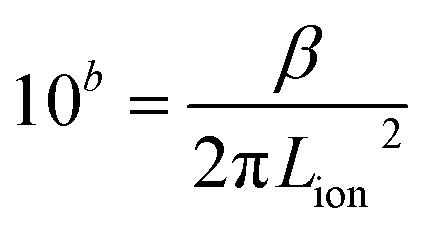


The fitted intercept implies an effective ionic diffusion length of approximately 200–600 nm for *β* = 0.1–1. This length scale is comparable to the film thickness (≈550 nm), supporting the hypothesis that ion migration across the bulk of the film contributes to the observed low-frequency IMPLS response and agreeing with our earlier report.^32^

## Conclusions

In conclusion, we demonstrated that the PL spectral components – specifically, the PL peak energy and FWHM – exhibit measurable responses under modulated excitation that are distinct from the corresponding PL intensity response. These spectral responses can be visualized on spectral Bode and Nyquist plots using the defined peak energy and FWHM transfer functions.

We also performed a simple compositional analysis to investigate the relative contributions of the halide species on the IMPLS response. Under full-field LED excitation, the observed reduction in IMPLS amplitude was associated with the iodide-containing compositions, whereas the pure-bromide sample exhibited a qualitatively different response within the measured frequency range. The approximate order-of-magnitude difference in onset frequencies between the pure-iodide and pure-bromide perovskites is consistent with the corresponding difference in their diffusion coefficients, supporting our previous hypothesis that the processes in both materials are diffusive in nature.^15,51,52^ From the high-intensity localized experiment using additional laser excitation, we found that the mixed-halide compositions exhibited stronger responses than the pure halides in terms of their spectral components, suggesting that these features may be influenced by interactions between the two halide species, including possible phase-segregation effects, rather than a single halide dominating the spectral signal.^42^ However, compositional information inferred from PL must be interpreted with caution, since the PL spectrum is also influenced by carrier funneling, defects, reabsorption, and other coupled optical and electronic processes. Further evidence for the compositional origin of the measured response will require correlating IMPLS measurements with X-ray fluorescence maps of the local composition, together with drift-diffusion modeling to isolate the contribution of mobile ions to the IMPLS response. These results will be presented by us in an immediate follow-up work.

As an outlook, we envision that quantifying the three separate IMPLS components could reduce model uncertainty in fitting procedures for optical equivalent circuit models and drift–diffusion simulations, analogous to the way impedance spectroscopy is typically combined with complementary electrical techniques.^12,16,58^ More broadly, IMPLS offers a powerful, contact-free diagnostic tool for assessing perovskite material quality while directly probing ionic processes. When combined with established optical methods such as time-resolved photoluminescence spectroscopy and photoluminescence quantum yield analysis, IMPLS provides complementary insights into the complex ionic–electronic interactions – including compositional insights from the spectral element – in halide perovskite films and perovskite solar cells. With further development, IMPLS could be integrated into pilot-line and manufacturing workflows to enable scalable quality control and guide the design of more stable, high-efficiency perovskite optoelectronic devices.

## Conflicts of interest

The authors declare the following competing interest: S. C. G., A. O. A., B. E., and E. C. G. have submitted a patent application for a modulated photoluminescence technique to characterize mobile ions. The authors declare no other conflicts of interest.

## Supplementary Material

EL-OLF-D6EL00090H-s001

## Data Availability

Raw data can be requested from the authors. The data supporting this article have been included as part of the supplementary information (SI). Supplementary information: materials, methods, supporting measurements. See DOI: https://doi.org/10.1039/d6el00090h.
