# Supplementary material for: Resolving mobile halide diffusion in perovskite films with frequency-domain photoluminescence
Source: EES Solar. 2026 Sep 10. Online ahead of print. doi: 10.1039/d6el00090h (PMC13591135; doi:10.1039/d6el00090h)
Supplement: Supplementary file 1 [file EL-OLF-D6EL00090H-s001.pdf]

Supplementary Information (SI) for EES Solar.
This journal is © The Royal Society of Chemistry 2026

Supporting Information
Resolving Mobile Halide Diffusion in Perovskite Films with Frequency-Domain Photoluminescence

Sarah C. Gillespie,[a] Agustin O. Alvarez,[a] Bruno Ehrler,[a] Veronique S. Gevaerts,[b] L.J. Geerligs,[b] Gianluca Coletti,[c] and Erik C. Garnett*[a,d]

[a] LMPV-Sustainable Energy Materials Department, AMOLF Institute, Science Park 104, Amsterdam, 1098XG, The Netherlands.
[b] TNO Department Solar Energy, Westerduinweg 3, Petten, 1755LE, The Netherlands.
[c] School of Photovoltaic and Renewable Energy Engineering, University of New South Wales, Sydney, Australia.
[d] University of Amsterdam, Science Park 904, Amsterdam, 1098XH, The Netherlands.

E-mail: e.garnett@amolf.nl

S1 Sample Fabrication

The triple-cation, mixed-halide (TCMH) perovskite was fabricated as described in our previous work[1]. To prepare the $MAPb(I_xBr_{1-x})_3$ films, under nitrogen conditions, PbI_2 (≥98%, TCI) and $PbBr_2$ (≥98%, TCI) were separately dissolved in 4:1 DMF:DMSO solvent mixtures (1.5 M) by stirring at 70 °C for three hours. The resulting PbI_2 and $PbBr_2$ solutions were then added to pre-weighed MAI (≥99%, TCI) and MABr (≥98%, TCI) precursors and further stirred (70 °C for two hours) to yield 1.24 M solutions of $MAPbI_3$ and $MAPbBr_3$, respectively. Parts of each solution were then combined in the appropriate ratios to prepare the $MAPb(I_{0.8}Br_{0.2})_3$ and $MAPb(I_{0.5}Br_{0.5})_3$ solutions, which were further stirred at 70 °C for one hour. The four solutions were then cooled to room temperature and filtered with 0.22 µm PTFE filters before spin coating.
Glass substrates were prepared by cleaning with 1% Hellmanex III solution in deionized (DI) water. They were then sonicated in 70 °C DI water (15 minutes), acetone (15 minutes) and finally isopropanol (15 minutes) before they were thoroughly dried under a high-pressure nitrogen gas flow. The substrates were further treated under UV-ozone for 45 minutes before they were immediately transferred to the glovebox. 120 µL of the perovskite solution was deposited on the glass substrates and then spin-coated at 5000 RPM for 30 seconds, with an 833 RPM/s ramp time. Halfway through the spin-coating procedure, the solution was quenched using 170 µL of filtered chlorobenzene. The perovskite films were then annealed at 100 °C for 20 minutes each. All films were then stored in the glovebox overnight before being encapsulated in 60 nm of SiO_2 by electron-beam evaporation (Polyteknik Flextura M508E) at 0.06 nm/s from an SiO_2 target. Immediately after encapsulation, the samples were characterized over two days and stored in the glovebox between measurements.

S2 Characterization of $MAPb(I_xBr_{1-x})_3$ Thin Films

X-ray diffraction (XRD; Bruker D2 Phaser with Cu Kα radiation) and absorbance spectra (PerkinElmer LAMBDA 750 UV/Vis/NIR spectrophotometer) were measured for the $MAPb(I_xBr_{1-x})_3$ samples to confirm material formation and to determine the band gaps[2]. The corresponding XRD patterns, absorbance spectra and instantaneous PL spectra are shown in Figure S1, with the extracted band gaps summarized in Table S1.

Table S1: Band gaps of $MAPb(I_xBr_{1-x})_3$ extracted from Tauc fitting of the absorbance data[2]. The uncertainties of the fitted band gaps are ±1 meV.

**Composition**	$MAPbI_3$	$MAPb(I_{0.8}Br_{0.2})_3$	$MAPb(I_{0.5}Br_{0.5})_3$	$MAPbBr_3$
**Band gap (eV)**	1.596	1.688	1.871	2.297

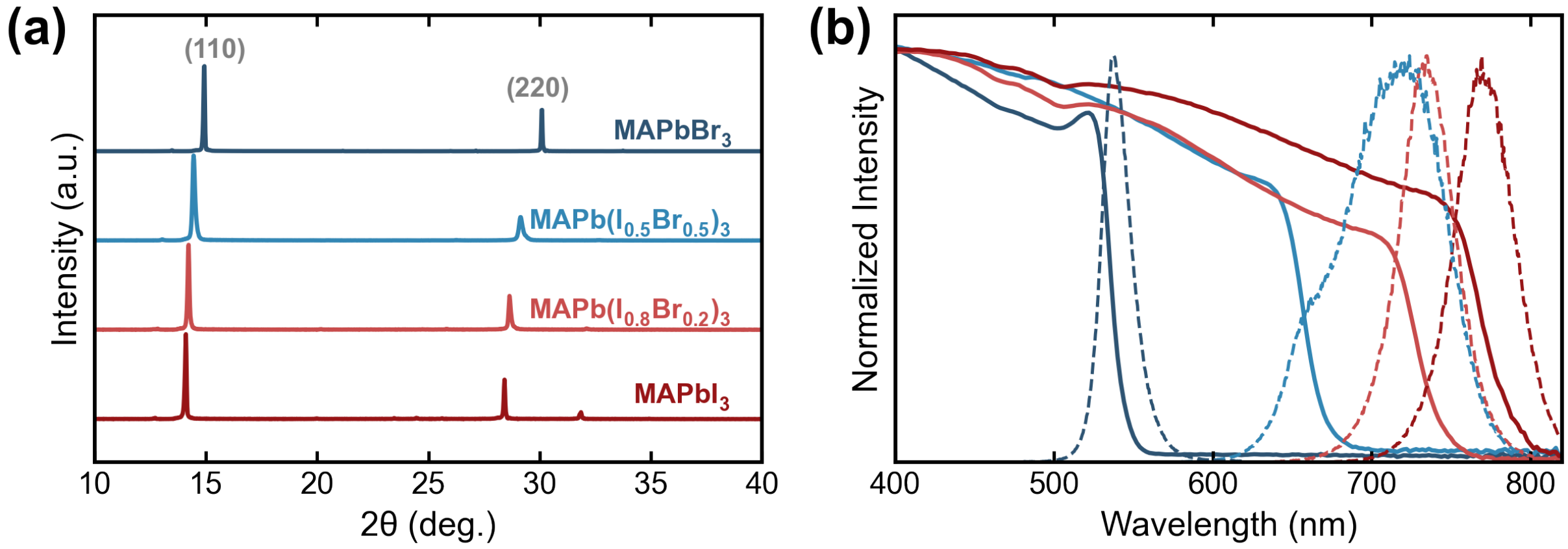

Figure S1: (a) XRD patterns of the $MAPb(I_xBr_{1-x})_3$ samples confirming perovskite formation. (b) Normalized absorbance spectra (solid curves) and PL spectra (dashed curves). The PL spectra were collected approximately 15 s after excitation was applied.

S3 IMPLS Experimental Details

In this work, IMPLS was first measured on the SiO_2-encapsulated TCMH perovskite film with the composition $Cs_{0.07}(FA_{0.8}MA_{0.2})_{0.93}Pb(I_{0.8}Br_{0.2})_3$. A schematic of the experimental setup is shown in our previous work[1]. This setup is comprised of a WITec alpha300 SR confocal imaging microscope, coupled to a ThorLAbs S1DC405 405 nm CW diode laser and to a Cree 450 nm LED. The LED intensity and sinusoidal modulation was driven with a 33522A arbitrary waveform generator (Keysight Technologies). The LED was positioned at the rear of the perovskite sample. The LED offset excitation intensity was set at 38.1 mW/cm^2, and the superimposed modulation amplitude was set at 15.7 mW/cm^2. In addition, a focused 405 nm laser (operating in reflection mode using a dichroic mirror) supplied localized excitation at 33.1 W/cm^2. The 405 nm laser was focused onto a spot area of 78.5 μm^2 using a 20× objective (Zeiss).
IMPLS was collected for nine frequencies between $f = 2$ mHz and 1 Hz. For each frequency, both the LED and PL spectra were collected over time using a charge-coupled device (CCD) and spectrometer. The measurements were collected on different spots on the sample, and the frequencies were applied in random order to prevent any sample hysteresis effects from accumulating during the experiment[3, 4]. As outlined in the main text, all spectra were fit to Lorentzian profiles. The integrated PL intensity, PL peak energy and PL full width at half maximum (FWHM) were determined from these fits. By tracking the integrated PL intensity, PL peak energy, and PL FWHM in time and fitting each of these traces to sine functions, the DC offsets, AC amplitudes, and phase shifts relative to the LED intensity were obtained. For the analysis, the response at each frequency was averaged over at least five stable modulation cycles, except at f = 2 mHz, for which one complete stable cycle was used because of the long measurement duration. The reported values were further averaged over at least three independent measurements.

IMPLS was then measured on the SiO_2-encapsulated $MAPb(I_xBr_{1-x})_3$ compositions using the same experimental setup as described above. For the measurements using both the LED and the laser (Figure 3 in the main text), the laser intensity was set at 2.74 W/cm^2. The approximate order-of-magnitude reduction in excitation intensity compared to the triple-cation mixed-halide measurements was necessary as photodegradation occurred in iodide-containing $MAPb(I_xBr_{1-x})_3$ samples at higher excitation levels.

Otherwise, the experimental conditions and protocols were kept the same as the conditions for the TCMH measurements. Figure 3 shows several deviations from otherwise smooth frequency-dependent trends. Similar deviations were observed in our previous IMPLS study, despite the use of denser frequency sampling[5]. These features may arise from multiple overlapping processes with similar characteristic timescales; however, the present frequency resolution is insufficient to distinguish them conclusively from experimental variation and noise.
For the IMPLS measurements which did not use the additional excitation from the laser (Figure 4 in the main text), the LED's DC offset intensity was set to 56.43 mW/cm^2, and the LED's amplitude was set to 30% of its DC offset intensity.

S4 Supporting Results

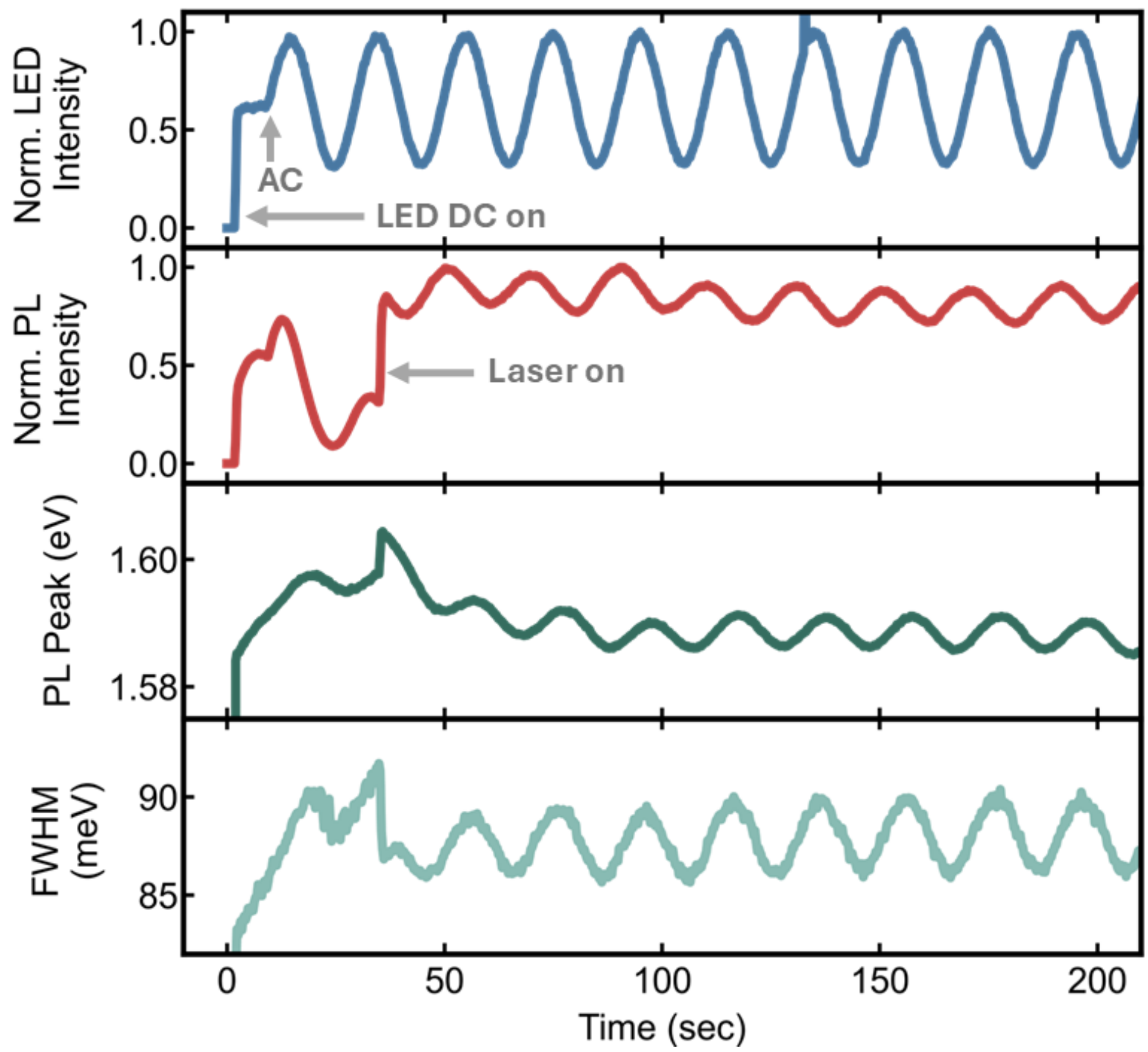

Figure S2: Extended time series of the experimental data shown in Figure 1c. The time points at which the LED DC illumination, LED AC modulation, and laser DC illumination were switched on are indicated by gray arrows. The PL intensity, peak energy, and FWHM stabilized after approximately 100 s of illumination; therefore, data analysis was performed only on measurements collected after this initial stabilization period.

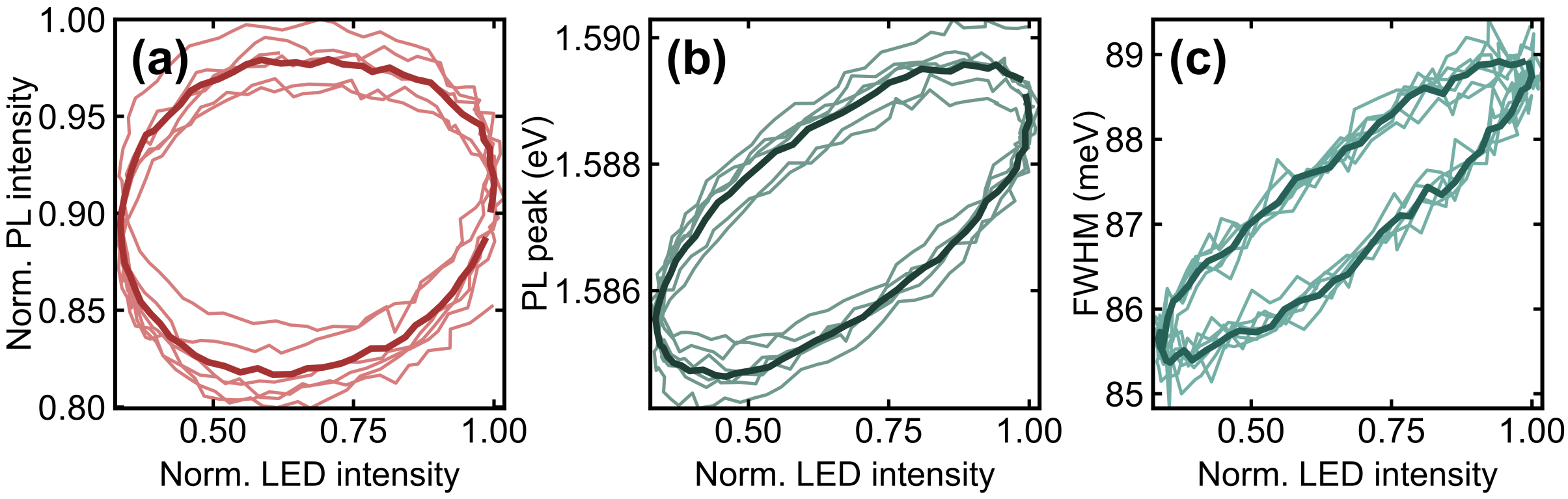

Figure S3: Lissajous plots at $f = 50$ mHz, corresponding to the data shown in Figure 1c, where (a) PL intensity, (b) PL peak energy, and (c) PL FWHM. The darker, thick line in each panel represents the mean cycle. All three plots exhibit elliptical behavior, indicating a linear response to the applied LED modulation.

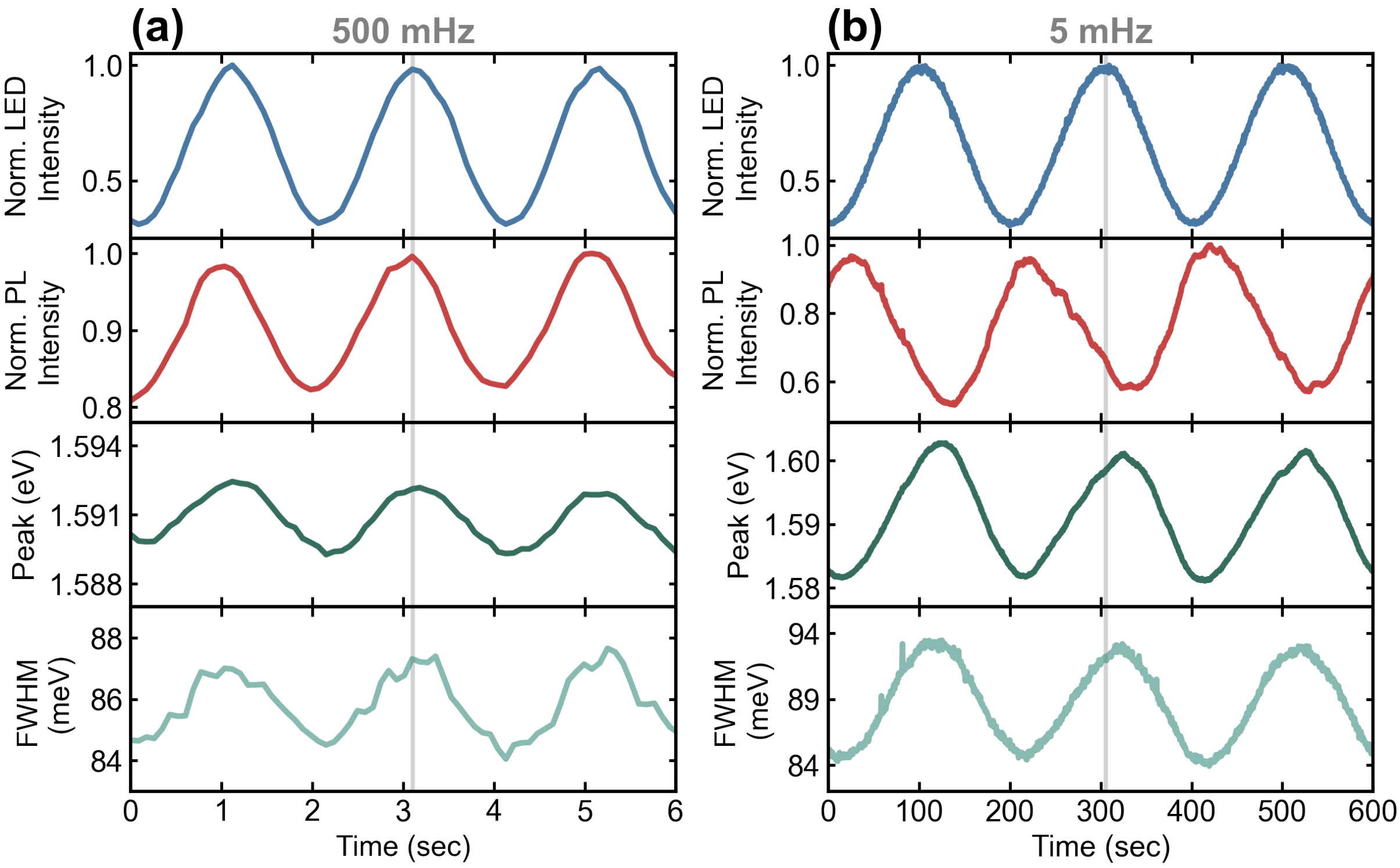

Figure S4: Time series at (a) $f = 500$ mHz (6 s), and (b) $f = 5$ mHz (600 s) of the integrated LED intensity (blue), integrated PL intensity (red), PL peak energy (dark green) and FWHM (light green). The gray solid line in both panels guides the eye to indicate frequency-dependent changes in the PL intensity and spectral responses.

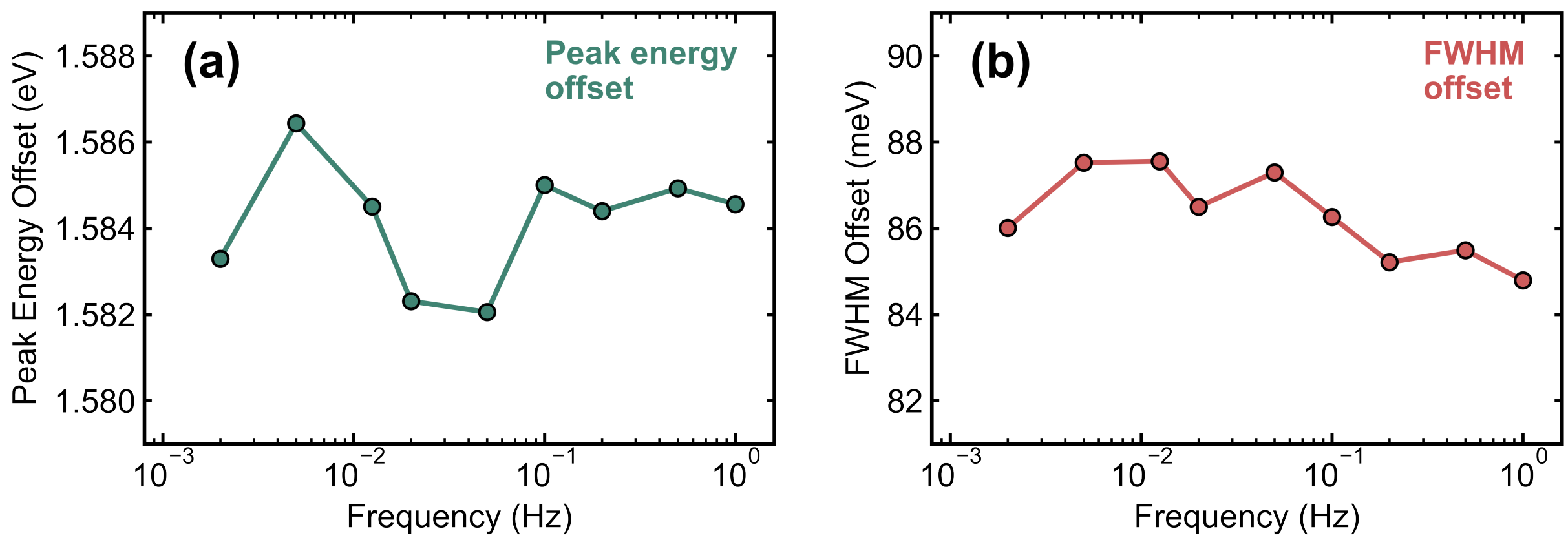

Figure S5: (a) Peak energy and (b) FWHM DC offsets for the TCMH perovskite sample corresponding to the results in Figure 2 of the main text.

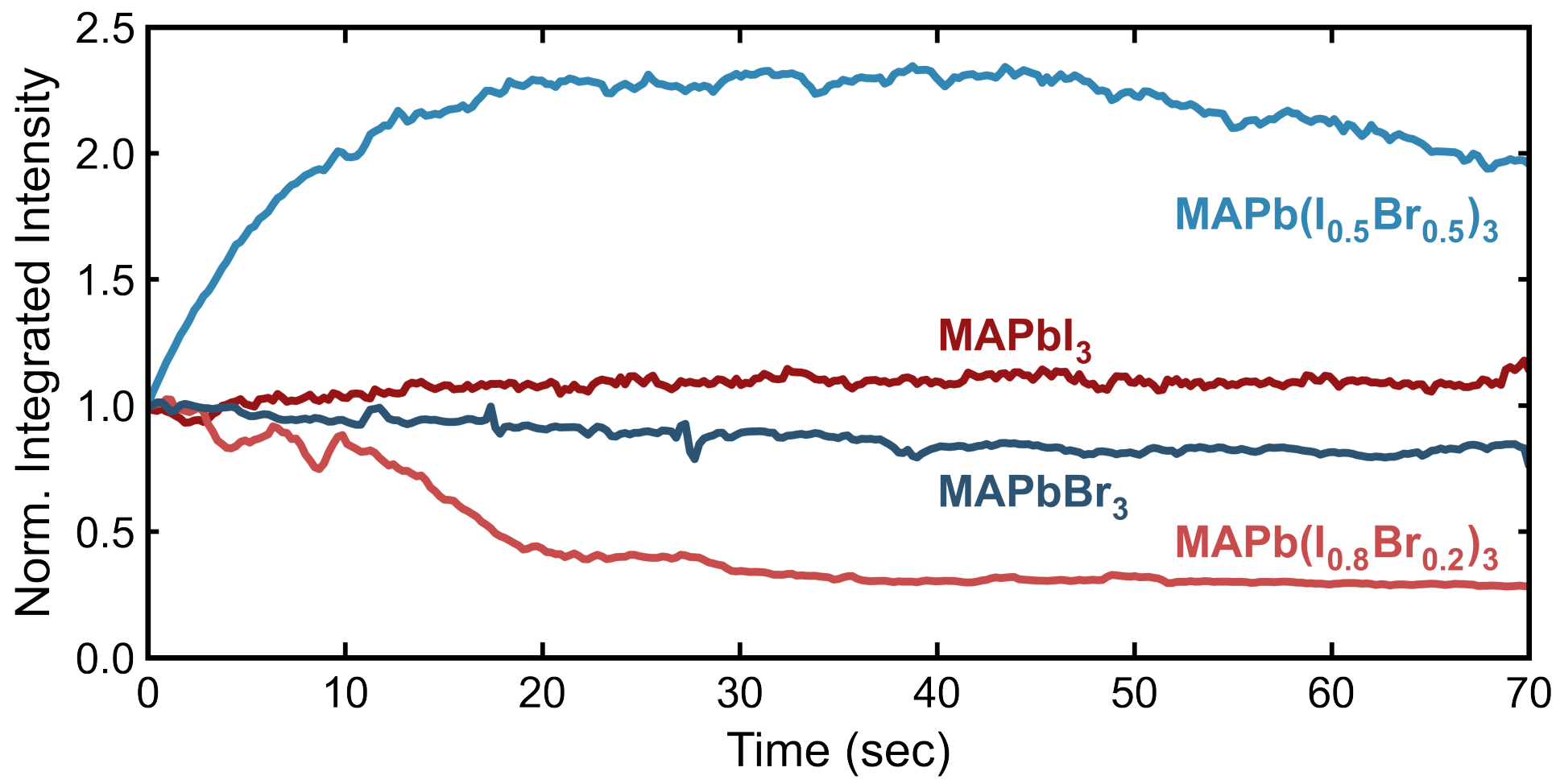

Figure S6: Integrated PL intensity time series for the four $MAPb(I_xBr_{1-x})_3$ samples, measured with a focused 405 nm laser (without LED excitation and without sinusoidal excitation modulation) at a DC excitation intensity of 2.74 W/cm^2

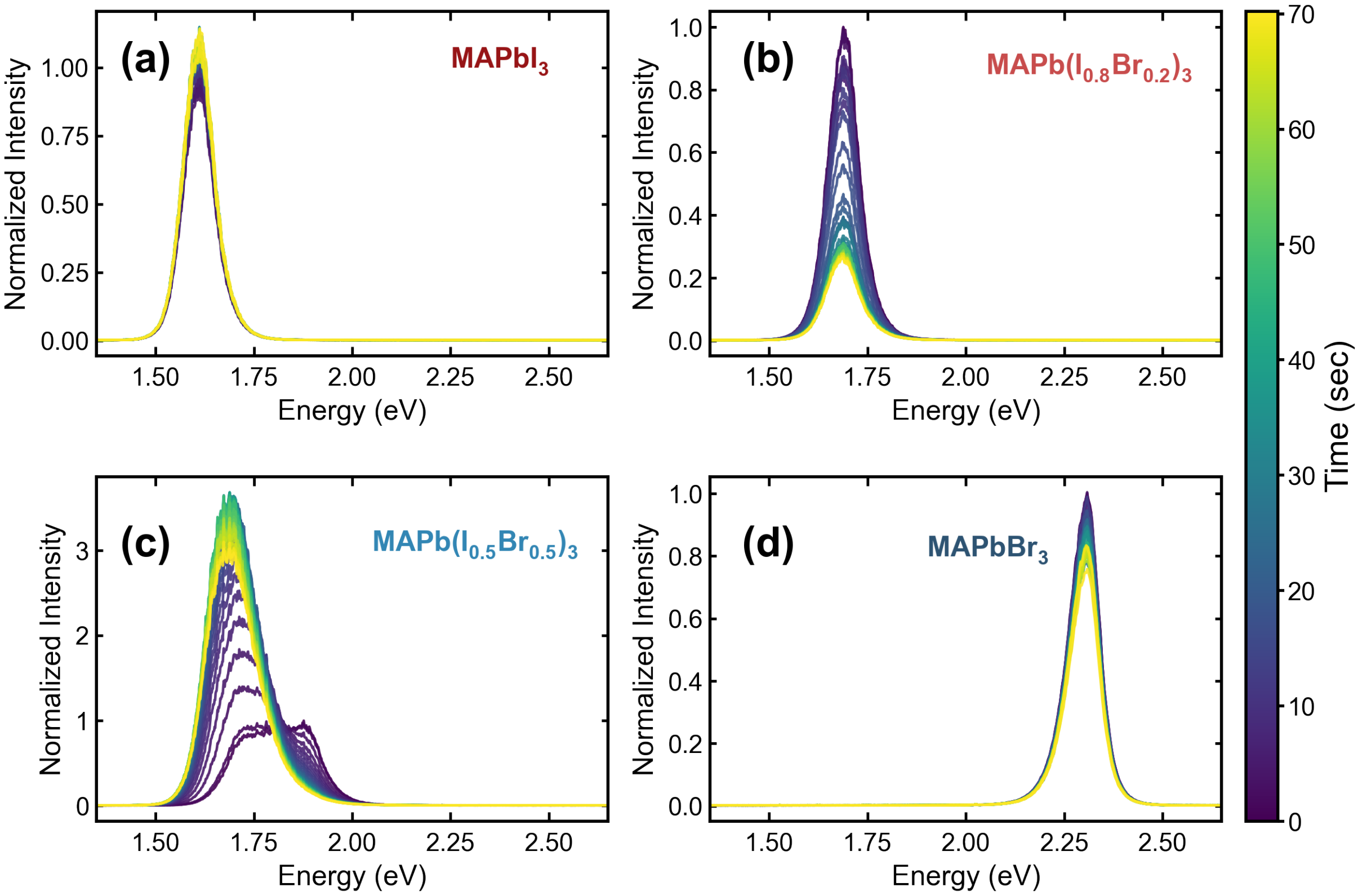

Figure S7: Stacked PL spectra (45 traces) of (a) $MAPbI_3$, (b) $MAPb(I_{0.8}Br_{0.2})_3$, (c) $MAPb(I_{0.5}Br_{0.5})_3$, and (d) $MAPbBr_3$ recorded between $t = 0$ and $t = 70$ s with an integration time of 83 ms. Spectra were obtained under 405 nm laser excitation only (without additional LED illumination or modulation).

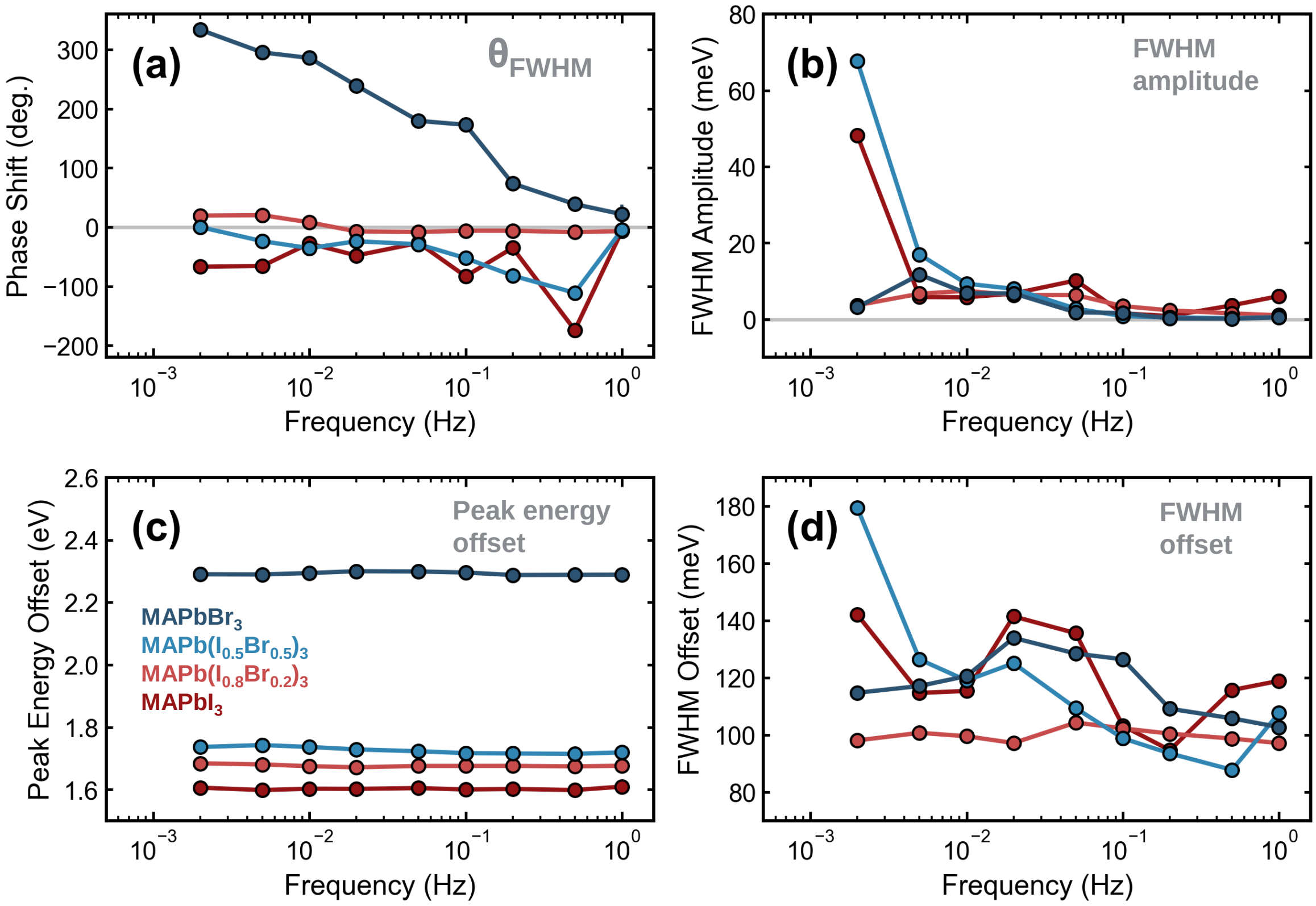

Figure S8: Supporting IMPLS results corresponding to the measurements shown in Figure 3 of the main text. (a) Phase shift and (b) amplitude of the FWHM for the four $MAPb(I_xBr_{1-x})_3$ perovskite compositions. (c) Peak energy DC offset and (d) FWHM DC offset for the same compositions.

References

[1] Sarah C. Gillespie, Jérôme Gautier, Linde M. van de Ven, Agustin O. Alvarez, Bruno Ehrler, L.J. Geerligs, Veronique S. Gevaerts, Gianluca Coletti, and Erik C. Garnett. Photoluminescence mapping of mobile and fixed defects in halide perovskite films. *ACS Energy Letters*, 11(4):3330–3339, 2026.

[2] Patrycja Makuła, Michał Pacia, and Wojciech Macyk. How to correctly determine the band gap energy of modified semiconductor photocatalysts based on uv–vis spectra. *The Journal of Physical Chemistry Letters*, 9(23):6814–6817, Dec 2018.

[3] Julia S. van der Burgt, Francesca Scalerandi, Jeroen J. de Boer, Susan A. Rigter, and Erik C. Garnett. Perovskite plasticity: Exploiting instability for self-optimized performance. *Advanced Functional Materials*, 32(39):2203771, 2022.

[4] Sarah C. Gillespie, Jarla Thiesbrummel, Veronique S. Gevaerts, L. J. Geerligs, Jeroen J. de Boer, Gianluca Coletti, and Erik C. Garnett. Excitation intervals enhance performance in perovskite solar cells. *ACS Applied Materials & Interfaces*, 17(43):59476–59485, 2025.

[5] Sarah C. Gillespie, Agustin O. Alvarez, Jarla Thiesbrummel, Veronique S. Gevaerts, L.J. Geerligs, Bruno Ehrler, Gianluca Coletti, and Erik C. Garnett. Intensity-modulated photoluminescence spectroscopy for revealing ionic processes in halide perovskites. *ACS Energy Letters*, 10(7):3122–3131, 2025.
